# Detection of Impending Aggressive Outbursts in Patients with Psychiatric Disorders: Violence Clues from Dogs

**DOI:** 10.1038/s41598-019-52940-w

**Published:** 2019-11-21

**Authors:** Uriel Bakeman, Hodaya Eilam, Clara Moray Schild, Dan Grinstein, Yuval Eshed, Morris Laster, Ester Fride, Sharon Anavi-Goffer

**Affiliations:** 1 Mind Print Ltd., 7/3 Yair Stern St., Herzeliya, 46421 Israel; 2 Unaffiliated, San Jose, California USA; 3 Forensic Psychiatry Inpatient Unit, Abarbanel Mental Health Center, Bat Yam, 59100 Israel; 4 Yoad Eshed Ltd., Bet-Yehoshua, 40591 Israel; 5 Institute of Medical Sciences, University of Aberdeen, Aberdeen, AB25 2ZD UK

**Keywords:** Brain-machine interface, Psychosis

## Abstract

Aggression in psychiatric wards is a continuing matter of concern for both patients and medical staff. Here we have tested the hypothesis that the frequency of such incidents can be reduced with a new strategy of using trained alert dogs that warn of impending violent outbursts. Dogs were positioned among patients in psychiatric wards. Analyses show that the dogs warned of impending aggressive outbursts, responding to signals from a specific patient out of a group of unfamiliar psychotic patients. Their alerts were not a response to stress as canine cortisol levels were not significantly changed. Visual glance was the preferred method used by young dogs to respond to patient. Until a similar electronic technology is developed, trained alert dogs can help caregivers to protect both the patient and those around them from injuries that may otherwise result from aggressive outbursts in psychiatric patients.

## Introduction

Aggression in psychiatric wards is a matter of concern for patients, their families and the medical staff. It has been reported that 8 to 44% of inpatients present violent behaviour during their stay in psychiatric wards and nearly 80% of the caregivers have experienced aggressive behaviour. Recent studies have suggested new strategies which may help the medical staff decrease the level of aggression in the ward^1^. In recent years, improving human well-being by animal-assisted therapy, especially by dog ‘therapists’ as companion animals, has been shown to be a successful approach that enhances the patient’s well-being^2,3^. Compared with ‘assistance dogs’, which are trained to work alongside an individual with a disability and to respond to clinical signs after onset^3,4^, ‘alert dogs’ demonstrate a characteristic, attention-seeking behaviour prior to the appearance of clinical signs^3,5–7^. Alert dogs can warn their leaders, either adults or children, of impending medical events such as epileptic^5,8–11^ or hypoglycaemic seizures^7,12–15^. Most studies so far indicate that a single dog can detect and alert of impending medical events in a single human being among a group of healthy humans^5,9,12,16,17^. However, it is not clear if dogs can alert of impending medical events in a single human being among a group of other humans diagnosed with a similar condition.

**Figure 1:**
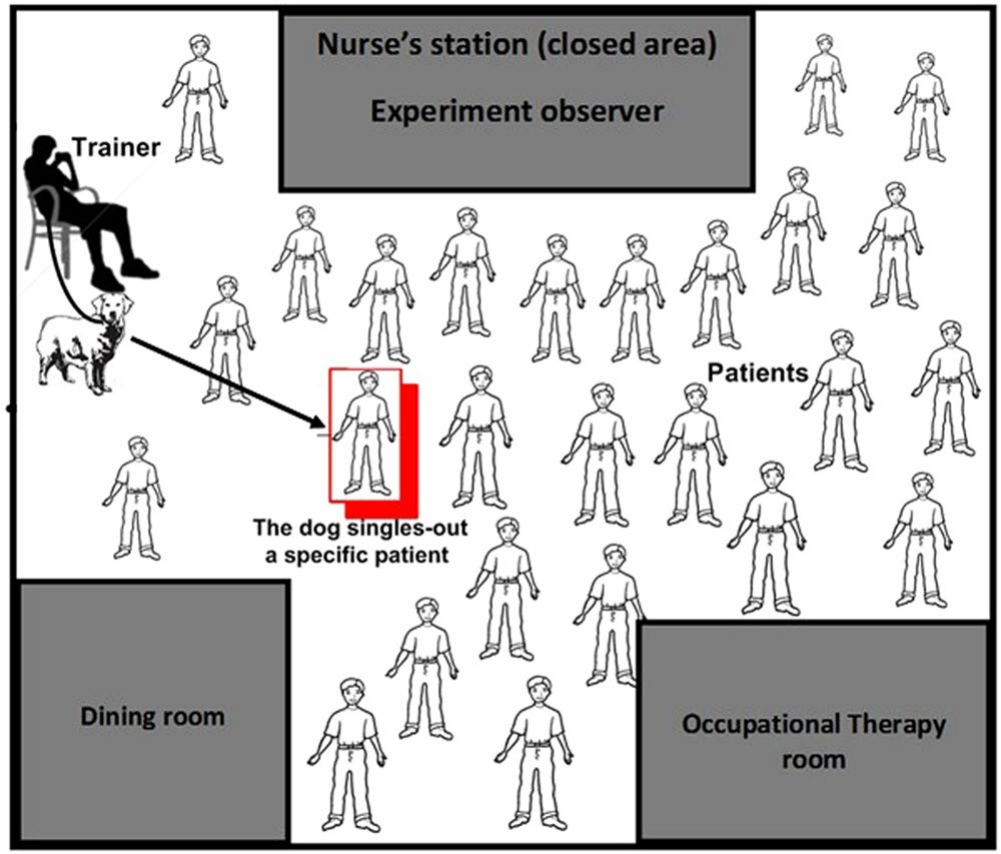
Schematic illustration of the visible area of the ward. During a session, the trainer was located at a fixed position in the ward (i.e. recreation area).

In this study we hypothesised that specially trained dogs can warn of violent outbursts of inpatients in psychiatric wards. This hypothesis is based on the integration of current knowledge from several reports including reports on the ability of pet dogs to anticipate physiological changes in patients^4,8–10,12,18,19^ or changes in human mood^6^, as well as on reports of other diseases (reviewed by^20^) that dogs can detect volatile compounds from the breath of patients with schizophrenia^21^, from stools/urine of patients with cancer^17,22^ and from other parts of the body. Taken together, novel aspects of this study were: (1) The dogs were expected to detect emotional or behavioural changes, which were initiated by change/s in the patient’s psycho-physiological state; the mechanism for these changes is not yet known, presumably emotional or behavioural changes underlie the first step in this chain reaction; (2) The dogs were expected to signal a specific patient who undergoes a change in the level of aggression out of a group of patients with schizophrenia and/or related disorders; (3) In our study the dogs were required to respond to unfamiliar patients (i.e. non-owners). As with a previously reported study of trained dogs that distinguish urine of a patient with bladder cancer out of several urine samples of healthy patients^17^, in both studies the samples were of unfamiliar humans (to the dogs). However, in our study all the subjects were patients and the dogs were required to alert impending cases (see Fig. 1 for exact location). In contrast, other studies reported alert dogs responding to impending diabetic or epileptic events of their owners^4,8–10,12,18,19^. In these studies, the alerts might be influenced by the close attachment between the owner and the dog^23^.

## Results

### Prediction of patients’ violent outbursts by specially trained alert dogs

**Figure 2:**
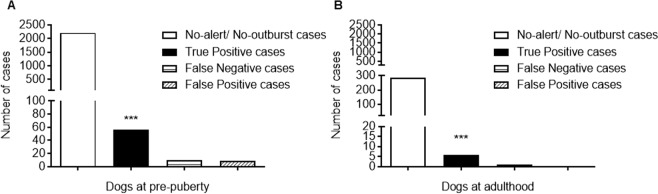
Trained-alert dogs identify violent outbursts in advance. A significant association was found between positive alerts and true outbursts when dogs were (**A**) at pre-puberty or (**B**) adult. ***p < 0.0001, Chi square (total N 293) or Fisher’s exact tests, respectively.

A significant association was found between positive alerts and true outbursts by pre-pubertal dogs (*p* < 0.0001, χ² = 1651, total frequencies = 2281, df = 1, two-sided; Fig. 2A; Table S2). The Chi square statistic indices for predictive accuracy demonstrated that out of 30 patients, who potentially could each have at least one violent outburst per day, 85% were positively alerted (Sensitivity, 95% CI 74.0–92.5%), while 99.6% of the patients who did not express violent outbursts were not alerted (Specificity, 95% CI 99.2–99.8%), predicting that 86% of the alerts will be followed by true outbursts (Positive Predictive Value, 95% CI 75.3–93.5%), leaving 14% cases as false-positive alerts, the Likelihood ratio was 208.8. The effect size values were: Attributable risk was 0.86 (95% CI 0.74–0.93), Relative Risk was 190.9 (95% CI 103.1–353.6), suggesting that a patient that had been alerted has had about 200% chance to outburst than a patient that had not been alerted. Odds ratio was 1373 (95% CI 523.2–3508), suggesting that the chances of a patient with violent outburst to be alerted is 1373 higher than the chances of a patient without outburst to be alerted.

In dog adulthood, there was a significant association between positive alerts (‘focused’) and true outbursts (*p* < 0.0001, Fisher’s exact test, total frequencies = 293, two-sided; Fig. 2B). The statistic indices for predictive accuracy demonstrate that out of 30 patients 85.7% were positively alerted (Sensitivity, 95% CI 42.1–99.6%), while 100% of the patients who did not express violent outbursts were not alerted (Specificity, 95% CI 98.7–100%). Thus, we can predict that 100% of the alerts by adult dogs will be followed by true outbursts (Positive Predictive Value, 95% CI 54.1–100%). The effect size values are: Attributable risk 0.99 (95% CI 0.51–1.01), Relative Risk was 287 (95% CI 211.9–1077), Odds ratio was 2483 (95% CI 92.16–66896).

The ‘effectiveness’ of the dogs as an alert under repeated observations per animal was tested. Two dogs took part in this study. Per dog, we examined whether alert rates changed over 170 hours (pre-puberty and adulthood) as dogs had time to habituate to the arena and this could affect their ‘effectiveness’. The Cochran’s Q test determined that there was no statistically significant difference in the proportion of alerts that the two dog exhibited over time, χ^2^(169) = 196.648, *p* = 0.072.

**Figure 3:**
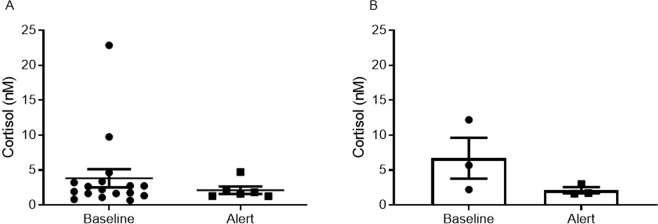
A comparison of cortisol level in dogs’ saliva before alerts and after alerts. A total of 24 samples were collected. (**A**) In the ‘baseline’ group, 18 samples were collected before the dogs started their sessions (17 samples analysed, one sample was below kit sensitivity). The mean cortisol level before alerts (‘Baseline’ group) was not significantly different from the mean cortisol level after alerts (‘Alert’ group). Results are expressed as means ± SEM, unpaired, two-tailed, Student’s *t* test *p* = 0.45. (**B**) Out of all samples, 3 pairs of samples (before/after alert) per each dog (a total of 6 pairs) were compared. Mean cortisol levels of the paired samples was not significantly different. Results are expressed as means ± SEM, two-way analyses of variance (ANOVA), matched values, *p* = 0.26, F(1,1) = 5.52.

The following results of parameters that reflect stress amongst others, further supporting that their signalling behaviour aimed to alert the trainer of impending outbursts: (1) A comparison of cortisol level revealed that there was no statistical difference between salivary cortisol level of the dogs before sessions started (‘baseline’) or after the alerts (Fig. 3A shows all samples, 3.83 ± 1.29 nM (n = 17) vs. 2.12 ± 0.53 nM (n = 6), respectively, *p* = 0.45, unpaired, two-tailed Student’s *t-test*; Fig. 3B shows 3 pairs of samples per each dog, two-way analyses of variance (ANOVA) of matched values, *p* = 0.26, F(1,1) = 5.52). This means that the ‘baseline’ cortisol level is similar to that previously reported (6 nM)^24^. These results further support that the dogs were not already aroused when arrived to the hospital, indicating that the dogs did not experience the situation as stressful; (2) In support of these results is the behaviour of adult dogs as documented in the video track: (a) The dogs did not escape from the ‘violent scene’ but appeared to pull towards it; (b) The dogs did not develop typical tension wounds; (c) The dogs did not pin their ears back, a behaviour that reflects anxiety.

### Comparison of number of outbursts with physical restraints

**Figure 4:**
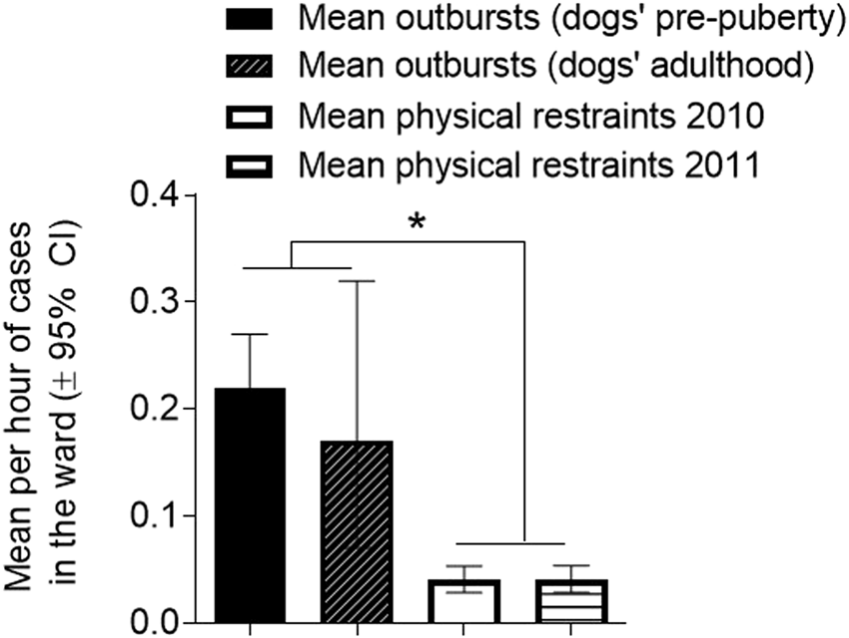
‘Outbursts’ vs. ‘Physical restraints’. The number of outbursts was significantly higher than the number of physical restraints at the wards. Results are expressed as means per hour (±95% CI). * *p* < 0.05 by confidence intervals.

**Figure 5:**
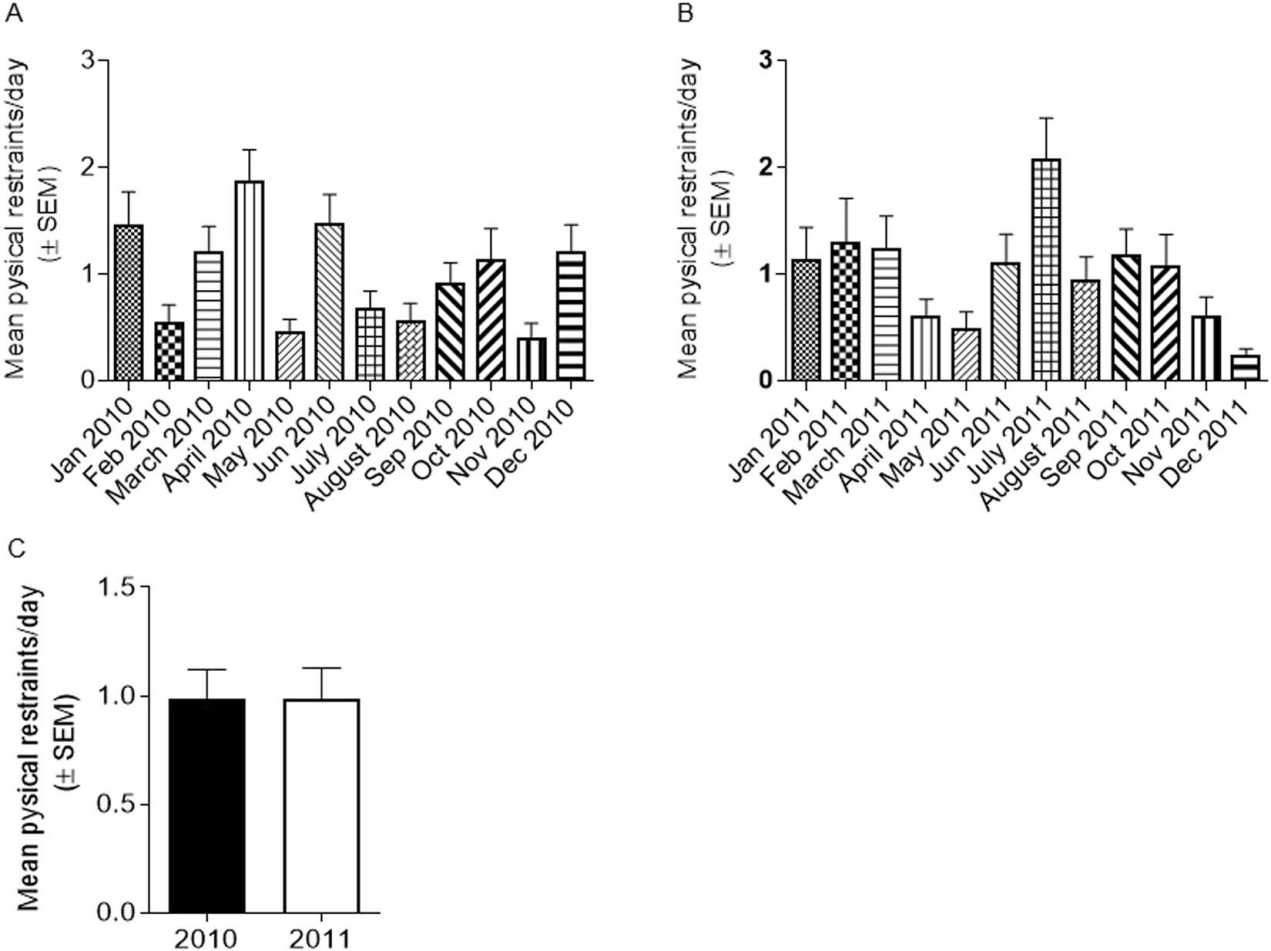
Physical restraints. The number of physical restraints per day in the ward for each month in the years (**A**) 2010 and (**B**) 2011. (**C**) The mean of physical restraints per day was calculated from the data in **A** and **B**.

We found that the mean of violent outbursts per hour in the wards (at dog pre-puberty or adulthood) was 4–5 times higher than the number of cases which ended with physical restraints per hour (*p* < 0.05, Figs 4 and 5). The mean of the number of physical restraints in 2010 was not significantly different from that in 2011 (Fig. 5C). The mean number of violent outbursts in the ward was not significantly different from each other in the two periods of the study (Fig. 4). These results further support the ability of the ‘alert dogs’ to alert impending violent outbursts.

### ‘Focused’ vs. ‘Unfocused’ behaviour: dogs identify outbursts at a distance

Alerts were divided into two main types of alert categories: ‘focused’ and ‘unfocused’ (Table 1). ‘Focused’ alerts were characterised by the ability of the dog to ‘mark’ a specific patient by staring at him. In addition, there were ‘unfocused’ alerts. These alerts were characterised by a change in the behaviour of the dog but without staring (e.g. pulling the leash or moving around; the full range of behaviour categories are listed in Table S3), suggesting an impending outburst but the trainer could not identify who is the specific patient that is expected to display anger.

**Table 1:** Canine alerting.

Age of dogs	Mean alerts/hour (95% CI)	Focused	Unfocused	Working hours	Range of alerts per session
Pre-puberty	0.25 (0.20–0.30)	65	11	304	1–2
Adult	0.39 (0.25–0.55)	7	7	36	2–4

**Figure 6:**
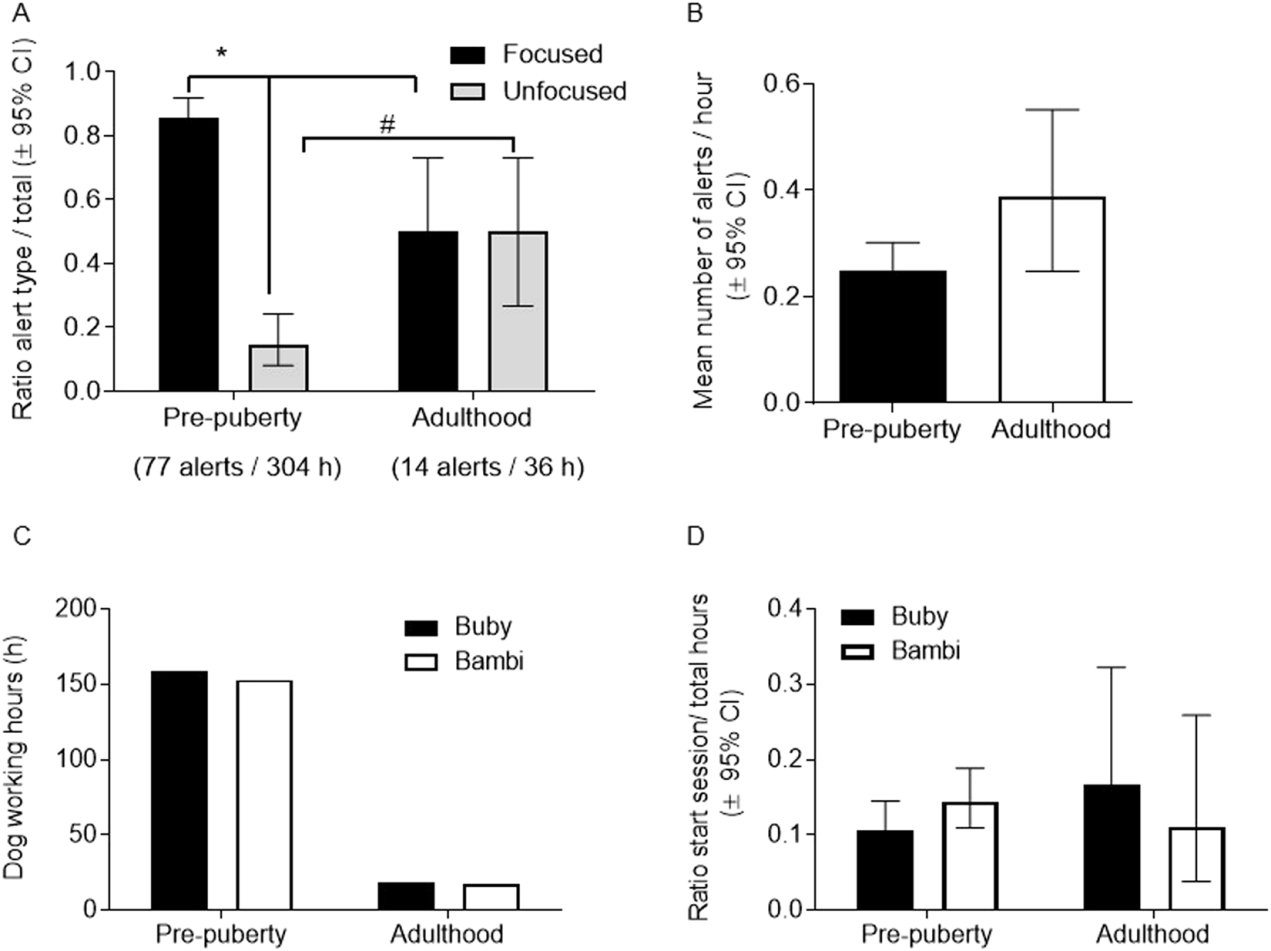
‘Focused’ vs. ‘Unfocused’ alerts. (**A**) Adult dogs produce significantly less ‘focused’ alerts and significantly more ‘unfocused’ alerts. (**B**) Adult dogs alert more frequently. (**C**) The number of working hours by each dog. (**D**) The frequency that each dog started a session. * vs. ‘focused’ and # vs. ‘unfocused’ at pre-puberty, *p* < 0.05, Wald method for 95% confidence intervals of ratios.

The relative ratios of ‘focused’ alerts vs. ‘unfocused’ alerts were calculated (±CI). Ratios of ‘focused’/‘unfocused’ alerts are presented in Fig. 6A.

The results show that at pre-puberty the relative proportion of ‘focused’ alerts was higher than at adulthood, while the relative proportion of ‘unfocused’ alerts at adulthood was higher than before puberty (Fig. 6A). The relative proportion of ‘focused’ alerts significantly (*p* < 0.05) dropped from 85.5% at pre-puberty (95% CI 75.7–91.9%; 65 focused alerts; 76 total alerts) to 50.0% at adulthood (95% CI 26.8–73.2%; 7 focused alerts; 14 total alerts), while the relative proportion of ‘unfocused’ alerts significantly (*p* < 0.05) increased from 14.5% at pre-puberty (95% CI 8.1–22.5%; 11 unfocused alerts; 76 total alerts) to 50.0% at adulthood (95% CI 26.8–73.2%; 7 unfocused alerts; 14 total alerts).

Importantly, cases that were marked as ‘unfocused alerts’ were further studied, because these cases may either be a ‘false-positive’ case or a positive event that took place in nearby wards that were not visible to the dogs (or to the trainer/observer). By correlation with the information from the hospital, we discovered that in the nearby wards there were cases of violence near to the time of the dog alert. These results suggest that a proportion of these ‘unfocused alerts’ were actually ‘true positive’ impending violent outbursts, which were identified by the dogs despite the lack of visibility. When the dogs were adult, we recorded 7 ‘unfocused’ alerts, of those 5 alerts corresponded to 3 events in adjacent wards, one alert corresponded to one event within the ward but in a room which was not visible to the dogs, one alert out of the 7 ‘unfocused’ alerts was marked as a false-positive alert because an indication for an outburst in the ward or in nearby wards was not founded. These corresponding events were well documented by the hospital, producing a success rate of 85% (95% CI 47–99%) correct alerting.

Thus at adulthood the number of ‘unfocused’ alerts significantly increased, suggesting that mature dogs need less to focus on the source of the alert as they spontaneously learn to communicate via alternative set of behaviours. They are also more proficient, presenting fewer False Positive alerts. In addition, the rate of False Negative alerts (missed outbursts) was reduced from 15% during pre-puberty to 11% during adulthood. Collectively, these results suggest that adult dogs may be preferable because they alert outbursts in time, reducing the risk of unnecessary treatment, but the isolation of a hostile patient will be preferable using young dogs which show higher rate of ‘focused’ alerts. Future research will need to study how trainers can improve the rate of ‘focused’ alerts. It should be considered that this may require allowing the dogs to freely walk towards the patient. In some settings, such as a psychiatric hospital, these alerts save lives.

### Visual glance is the preferred method used by young dogs to respond to patient

We further report the contribution of visual cues. The dogs and the trainer were positioned in a closed room without patients present. The results show that in the first session there were no alerts and no outbursts (a session of 2 hours). In the second session there were 3 ‘unfocused’ alerts, for one of which the dog was facing towards another department (a session of 4 hours, 3 days after the first session). The observer reported on several patients in the hall of whom one patient refused to receive treatment and another patient shouted (within the hall). During the third session the dog Buby became alerted and faced towards another department (a session of 2 hours, 7 days after the second session). We have tracked this event at the other department. At 20:00 pm (end of visiting hours), 3.5 hours after Buby’s alert, a patient attacked visitors. In the closed room, 4 ‘unfocused’ alerts were recorded, of those 2 alerts corresponded to 2 events producing a success rate of 50% (95% CI 15–85%) correct alerting. Although we cannot preclude responses to trainer’s cues, these results further support reporting the data (Fig. 6A) from ‘unfocused’ alerts.

In addition, the mean number of alerts per hour in the ward was not significantly different but showed a trend of increase when the dogs were adults (Fig. 6B). The increased number of alerts during adulthood can be attributed to the age of the dogs, their cognitive development, their experience, leading to higher skills under the same environment, or can be correlated to the number of violent outbursts per hour. There was a trend of increase in the outbursts per hour when the dogs were adult (average 0.17 for pre-puberty vs. 0.22 for adulthood). This trend could not be attributed to a specific dog as each dog worked a similar number of days per month and a similar number of hours (Fig. 6C, Table 2). There was also no difference between the times each dog started the sessions at adulthood (Fig. 6D).

**Table 2:** Violent outbursts in the visible area of the Dual Diagnosis ward and Forensic Unit.

Age of dogs	Mean outbursts/hour (95% CI)	Outbursts in the ward	Working hours	Days	Months	Days/month
Pre-puberty	0.22 (0.17–0.27)	66	304	77	7	11
Adult	0.17 (0.07–0.32)	6	36	10	1	10

The number of violent outbursts represents the number of outbursts within the confines of the ward (i.e. area which the dogs could visualise, Fig. 1). The number of days represents the number of sessions as one session was conducted per day. Most sessions lasted 4 hours.

In this study we noticed that the majority of the alerts were in the range of 4–30 min but occasionally the alerts started a few hours before the outburst. A minor number of cases of late events that preceded ‘focused’ alert at a specific patient in the ward were linked, as based on a pervious study^6^ our hypothesis assumed these are ‘true-positive’ cases, but events (early or late) without specific alerts were regarded as ‘false-negative’ whereas alerts without specific events were regarded as ‘false-positive’. Therefore, the time between the alert to the event was monitored, the second stage of this study, at adulthood. Out of 9 events, 4 events were firstly identified 4–10 min before the outbursts and 2 events were identified 10–30 min before the outbursts (by either ‘focused’ or ‘unfocused’ alerts), showing that over 50% of the cases were identified close to the time of the event (Table S4, repeated alerts were excluded from the analysis). However, it is worth to note that in both research periods we noticed that some cases were alerted by the dogs at an interval of hours. Linking alerts with events many hours later still requires further research. It will be important to examine if the size of the delay between the alert and event has an impact on the reliability of the prediction. Similarly, it is of interest to examine whether the distance between distant wards and the dog impacted the reliability of the prediction.

### Different dogs can alert the same patient vs. multiple alerts by a dog at the same patient

Considering the applicability of day-to-day support by dogs in psychiatric wards there is a need to take into account that due to regular circumstances (e.g. tiredness/changing shifts) or irregular circumstances (e.g. the dog is injured/sick) a dog will need to be replaced. In other words, would the replacement dog also identify the same patient as its predecessor? Supplementary Tables S5 and S6 demonstrate two examples showing alerting of a specific patient by two different adult dogs that were positioned at the ward sequentially. In the first case (Table S5), the dog Buby made a ‘focused’ alert on patient #1 and when replaced with Bambi, the second dog also identified a ‘focused’ alert on the same patient (#1). In the second case (Table S6, examples are shown in the video film), Buby was rotating, focusing on patient #2, who was also identified by the second dog. Evidence for the existence of multiple alerts at the same patient with a violent outburst was also found (4 occasions out of 7 ‘focused’ alerts at adulthood; 95% CI 24.9–84.2%). For example, Table S6 demonstrates that the dog Buby produced several ‘focused’ alerts on patient #2 within one session. Multiple alerts by the same dog that were followed by one outburst were recorded as one true event.

### Case report

There were two special cases in which the dogs alerted patients who committed/attempted to commit suicide. The first case happened when the dogs were at pre-puberty. The dogs signalled to their handler definitive robust ‘unfocused’ alerts during the entire session. More specifically, the dogs were unsettled and strongly pulled the leash towards another building. Two days later, a patient in that building committed suicide. The hospital reported that this patient was very distressed and that this behaviour started on the same day the dog alerted. This ‘death’ alert has not been recorded as the video was installed only when the dogs were adult and because we did not anticipate that to happen. Therefore, a similar behaviour is shown in the video around time 5:54 (titled: “behaviour similar to death alert”). However, here it was clear that the dog needed to defecate and was taken out. This behaviour was not counted as an alert. In the real case, the dog did not defecate though this might occur. The second case occurred when the dogs were adult. Both dogs produced ‘unfocused’ alerts towards another building. At 8:20 am, Buby alerted and when the dogs were swapped, Bambi alerted at 8:45 am. At 8:49 am screams from a patient were heard from that building and hospital staff were alerted to help with a patient who attempted to commit suicide.

## Discussion

The results of this study show that specifically trained dogs possess the ability to alert their handler of impending violent outbursts in a random group of unfamiliar, psychotic patients. The success rate of positively identified outbursts was 85% and 86% when the dogs were pre-puberty or adults, respectively. The result of Cochran’s Q test pointed that there was no statistically significant difference in the proportion of alerts that the two dog exhibited over time. Collectively, these results suggest that the dogs maintained their alerting skills with age and across sessions. A similar alerting-rate has been reported with trained, seizure-responsive dogs which started to alert their owner spontaneously^6^ and with specifically trained seizure-alert dogs^5^. It is not clear what underlies the mechanism which promoted the dogs to continue to alert on violent outbursts throughout the study if there was no immediate reward. However, it is possible that the process of alerting itself may serve as a reward to the dogs, in that the dogs were pleased they had managed to protect their ‘pack leader’ (the trainer/handler). In support of this hypothesis are the following studies: (1) the documented adaptive cooperative behaviour among unrelated humans during wars, specifically, that of soldiers defending their group and commander^25^; (2) results of a recent study showing that the dogs’ latency to detect explosives is depended on the level of anxiety of their handler^26^; (3) dogs need a reward^27^ but can continue to alert their owner without a reward^5,7–15^; (4) dogs consider the human in their ‘pack’^28^. Collectively, our study further supports that the psychological behaviour of alerting dogs might, at least in part, resemble altruism behaviour seen for humans. In further support of this idea is the evidence for: (1) altruism across species for example: apes and birds^29^; (2) altruism behaviour between unrelated species or unrelated animals including between human and canine^27,30,31^. Furthermore, gene polymorphism in the oxytocin system has been suggested to contribute towards altruism^32^. Though differences between studies may result from different methods used to select the dogs, this suggests the following idea: that the selection of superior altruistic alerting-dogs can be further improved by combining a genetic selection based on ‘altruistic’ genes with the ‘Tests of Campbell’^33–35^.

Importantly, compared with previous studies, which reported that a single dog can alert abnormalities in a single human being^4,8–10,12,18^, our study found that a single dog can alert a number of patients. We have also found that different dogs can separately alert the same patient, after the dog was swapped during an impending violent case. This is of importance as it means that the hospital does not need to be dependent on one dog. It should be noted that in this study the dogs were trained by a man and their alerting behaviour was studied only among male psychotic patients. As dogs are known to respond differently to men and women^36^, it will be useful to compare in the future their alerting success rate of impending violent outbursts among female psychotic patients.

We did not observe, at any age, any aggressive behaviour by any of the trained dogs towards the patients, caregivers or their handler, supporting the conclusions of Strong & Brown (2000)^37^ that trained-alert dogs do not exhibit adverse behaviour. The personal impression of Dr Schild was that the patients and staff greatly welcomed the dogs. Similar results were found among owners of alert dogs for Type I diabetes, demonstrating that the patients trusted their dogs and that alert dog ownership has improved their life quality^19^.

A key question for future research is what are mechanisms that underlie these alerts? Our results are similar to previous studies which reported that the time between dog alert and onset of clinical symptoms was between 30 sec to 45 min^5,6^, but could start as early as 3 hours before the clinical onset^6^. However, further research is needed to statistically link a dog alert with an impending violent case many hours later. The differences in time may reflect a specific cue involved in the process of detection by the dogs, which can also be related on a specific clinical disease. Rooney *et al*. also noticed that once the dogs start to alert in practice they appear to improve their own skills in a natural way, responding to new cues such as subtle changes in owners’ mood or behaviour^19^. Alternatively, differences may be attributed to the training methods. A recent study concluded that ‘alert dogs’ are not reliable to alert hypoglycaemic events due to high false-positive rate, however, each of the dogs was trained in a different way^38^.

Here, the dogs often communicated by long period of staring, suggesting that like most dogs, these ‘alert dogs’ have used mainly visual cues to detect changes. These cues may be involved in higher cognition processes which reflect the ability of dogs to inspect social interaction. Recent reports suggest that, mechanistically at least, discriminative processes of social gestures may be similar in human and dog brains^39–41^. However, we clearly observed ‘unfocused’ alerts and were surprised to find that these were correlated with events in other wards at the hospital, supporting that trained-alert dogs use additional cues, such as, sound or smell. Indeed, a recent study has demonstrated that interpretation of human gestures by dogs is regulated by human’s tone of voice^42^. Moreover, an increase of dogs’ searching behaviour was evident following correct detection^42^. It has also been documented that dogs can smell volatile compounds produced by tumours, assisting the detection of cancers^16,17^ and EEG recordings from epileptic patients show the existence of low frequency waves distributed across the brain hemispheres that can potentially be detected by dogs^4^. It is interesting to note that other studies suggest that complex partial seizures and mood changes, especially aggression and anxiety, share a similar mechanism in which neuronal excitability emerges originates in the amygdala, a brain area which controls emotional behaviour (reviewed by^43^).

Importantly, based on recent developments which use brain-derived motor signals to control electronic and virtual devices, a recent study suggested that two rats can couple their brains to convey brain-to-brain sensorimotor information, providing evidence for a novel mechanism of ‘super sense’ information transmission between animals^39^. This mechanism of brain-to-brain interface is believed to be a form of electrical current or even photostimulation generated by special channels or an unidentified form of neuronal communication, yet to be discovered^44^. Taken together with our study, it is possible that the proposed new mechanism underlies also animal-human brain’s coupling, specifically to our study, brain-to-brain transmission of human brain-derived emotional signals that are decoded by a specially trained dog as emotional instability. Our study proposes a new model system to further elucidate the nature of novel brain-to-brain communication cues. Future studies aim to specifically explore the mechanisms may underlie the different alerting modes/behaviours and dissect the cues needed for each type of alert.

In summary, this study reports that dogs can successfully serve as ‘alert dogs’ of impending violent outbursts in psychiatric wards that accommodate potentially violent patients. An effective warning of violent outbursts would enable the advanced isolation of the hostile person out of a random group of patients, protecting the remaining individuals but also protecting the hostile person from self-harm. Standardization of the specific protocols of dog training for each clinical condition can greatly improve this tool in order to achieve high rate of positive alerts.

## Methods

### Participants

Patients participating in this study were inpatients of the Dual Diagnosis Ward and the Forensic Psychiatry Inpatient Unit, Abarbanel Mental Health Center, Bat Yam, Israel. The study was approved by the Institutional Review Board (IRB) of Abarbanel Mental Health Center, Israel, Trial Registration 920090532, that is authorised and under the supervision of the Israel Ministry of Health (in accordance with the IACUC guidelines). Data were analysed anonymously. The study was conducted from November 2009 to June 2011.

The wards accommodate an average of 30 adult male patients (age 18+) at any one time, of which 25 are from the Dual Diagnosis Ward. Inclusion criteria: subjects were clinically diagnosed by the heads of wards as having psychotic disorders according to psychiatric evaluations of Structured Clinical Interview for Diagnostic and Statistical Manual of Mental Disorders (DSM)-4-TR (SCID). Of the Dual Diagnosis Ward: 75% of the patients were diagnosed with schizophrenia (co-occurring with substance use disorder), 8–10% with manic-depressive disorder and 13–15% with substance induced-psychosis and a personality disorder. Of the Forensic Psychiatry Inpatient Unit: 80% of the patients were diagnosed with schizophrenia and co-occurring substance use disorder and 20% with personality disorder.

The study protocol maintained the patient’s standard clinical setting including standard psychiatric medications, psychotherapies etc. Information regarding the patients was obtained from the patients’ medical files and from the clinical staff. The Israel Ministry of Health approved the IRB Committee of Abarbanel Hospital decision that the dogs were to be considered as a Bio-Medical Device. The dogs were held with a leash by their trainer and owner, Mr Bakeman, at all times.

Mr Bakeman provided his professional trainer certificate and provided evidence for the beneficial effects of alert dogs on patients (e.g. diabetics). The IRB Committee categorised this study as an observational study that involves intervention in the subjects’ environment. Accordingly, the IRB concluded that there was no need to require a written informed consent from each patient and member of staff, but each would provide a verbal consent.

A record of verbal consent was not required. For the Trial Registration 920090532 the IRB Committee of Abarbanel Hospital and the Israel Ministry of Health approved this consent procedure. Yet, the IRB ordered that for this study the Principal Medical Supervisor, Dr Clara Moray Schild (M.D.), would supervise actions to explain to the patients and staff about the study. This was done as a group during the weekly meetings the patients take in the department (by Dr Delayahu who has been supervised by Dr Schild) or to each individual patient (by Dr Schild). Dr Schild explained to the patients that the aim of the research is to identify violent acts and to prevent violence between patients. Although this explanation may have affected patient behaviour, violent acts still occurred. According to the decision of the IRB Committee, a patient who asked not to be included in the study was provided by Dr Schild with an alternative solution for the time the dogs were at the department.

### Patient daily behaviour report

A daily update was received from the medical staff regarding the mental state and behaviour of selected patients in the ward. We also analysed the hospital reports for the number of physical restraint events during years 2010 and 2011. However, as not all cases ended with a physical restraint, this was recorded only as a relative index of violent activity.

Control. The observers filled out an independent report at each session which documented: (1) visualised unusual/violent behaviour of patients; (2) a complementary report on behaviour of patients according to the hospital reports. The observer collected the hospital reports for up to 24 hours after each session; the reports were forwarded directly to the principal investigator of this study.

### Violent activity questionnaires

A hospital report was completed by the medical staff for each act of violence a patient exhibited. The report described the outburst details including suspected cause, time, severity, medical treatment outcomes. These reports were filled out daily. Table S1 outlines categories for violent outbursts according to nature and severity. The cut-off period for collection of the information was 24 hours after a session: (1) This was based on a previous study that recorded positive alerts even 3 hours before onset of clinical signs^6^, suggesting alert can occur at least a few hours before the event; (2) Our hypothesis was that every alert is a true alert. This means that alerts which were not followed by relatively immediate outbursts had to be followed; (3) The hospital reports (see below) were provided twice a day (morning and evening). The sessions were conducted at different hours along the day according to availability of the trainer and dogs. In order to standardise the data collection we selected a 24 hours cycle; (4) Extending this period also allowed to accommodate the possibility that different mechanisms may be involved in detecting the outbursts, for example: smelling volatiles, which are assumed to be released closely to the time of the event, but, may also be evoked many hours before the event; detecting brain waves, which may precede olfactory cues and, in theory, could be evoked by the patients many hours before events commence. Though studying the methods of detections was not in the scope of this study, the selected settings will promote studying these cues in the future.

### Please see Supplementary Information for a full description of Methods of ‘Training Programme of the dogs’ and ‘Behaviour questionnaire and evaluation of dog’s performance’

Briefly:

Step 1: Two Golden Retriever litter mate female pups were selected according to the ‘Tests of Campbell’ method^33,34^.

Step 2: At the age of 6+ weeks, the pups underwent behavioural analysis tests and initial training to determine their ability for showing general alerting responses.

Step 3: From the age of 2+ months the animals underwent a 30 day obedience training program, during which preliminary obedience skills (common commands: sit, down, stay, halt) were instilled through an operant conditioning method of positive reinforcement reward (pet/food/happy tones).

Step 4: In addition, for this study, the dogs underwent response training such as training for specific barks on command and activation of responses to emergency situations.

At the hospital: Once the dogs reached the age of two months they were also introduced to the hospital inpatient psychiatric ward for further training. At this point, the dogs were trained to bark when a patient outburst occurred. Importantly, in practice, when this training step was successfully completed, the dogs started to alert on impending events (see Results and Table S4- most cases were alerted 30 minutes prior to event).

The experiment began when both Golden Retriever dogs were introduced to the hospital ward from age 2 months up to 1.5 years and again at age 2 years. The number of observation days per month was similar between the two periods of the study (Table 2).

Their trainer was present at the ward for each session. Most sessions lasted 4 hours and only one session was conducted per day, after each session the dogs and owner left the hospital ground. Two dogs were used, each dog worked for 45 min alternating periods separated by periods of 1 hour and 15 min to rest and access to water.

The trainer sat on a chair during each work period (45 min) so that the trainer and the dog both had the same visual access to the patients, they were not separated from the patients and potentially could detect auditory and olfactory signals from the patients (Fig. 1). Also the patients had visual access to the dogs but, though petting was not allowed, we cannot exclude the possibility that the dogs somehow influenced the behaviour of the patients. In many cases the dogs could not visualise subtle facial movements (cues) of the trainer. Furthermore, most outbursts occurred some minutes and even hours after the alerts (see Table S4), suggesting that in these cases the trainer would not be in a position to transmit specific cues to the dogs.

In addition, our report identified whether the alert was directed at a specific patient or not; these alerts were termed ‘focused’ and ‘unfocused’ alerts, respectively.

### Video film of the alert dogs

Video recording of the dogs’ behaviour was approved by the IRB Committee of Abarbanel Hospital. Further to the ethical statements above, the IRB Committee of Abarbanel Hospital approved the final edited video for publication without a written or verbal consent under Trial Registration 920090532. According to the IRB Committee decision, the face of each patient was blurred before allowing the video to leave the hospital. Dr Schild approved that copy and Dr Anavi-Goffer further edited the video. The final version was approved for publication by the IRB Committee of Abarbanel Hospital. Author UB, dog handler, approved the video with a written consent. The video is available via Supplementary Information.

A video film was taken at dog adulthood to demonstrate the dogs’ behaviour following/at the time of different types of alerts, their behaviour at periods with no alerts and the interaction of the patients with the dogs (petting etc.). The video film suggests that the baseline behaviour of the dogs in the same environment without outbursts was laying on the floor, very often in a resting position. Note: There is a gap of 10 min between the real time vs. the time noted on the video. The video time was 10 minutes faster than the actual time.

### Physical restraints vs. violent outbursts

The wards recorded an average of 0.99 ± 0.095 (SEM, n = 24) physical restraints per day during the years 2010–2011 (Fig. 6A,B). As our trial records were on an hourly basis, dividing by 24, we calculated the mean of the number of physical restraints per hour. In our study, a total of 66 outbursts were recorded in the ward during the first period of study, when the dogs were at pre-puberty, with 304 observation hours. Mean violent outbursts per hour was 0.22 (0.17–0.27, 95% CI). Similarly, when the dogs were adults, the mean of violent outbursts per hour was 0.17 (0.07–0.32, 95% CI). These means of violent outbursts per hour were not significantly different. There was no access to the hospital reports apart for the time the dogs worked. Therefore, we could not calculate the mean of general violent outbursts per hour.

### Number of outbursts vs. alerts

At dog pre-puberty, 66 violent outbursts were recorded, of which 56 outbursts were alerted by the dogs (True Positive, TP) and 10 outbursts were missed, i.e. the dogs did not alert true outbursts (False Negative; FN) (Table S2). There were 9 alerts without recorded outbursts, i.e. there were no violent outbursts up to 24 hours after changes in dogs’ behaviour were recorded (i.e. False Positive; FP). At dog adulthood, 7 violent outbursts were recorded, of which 6 events were alerted by the dogs (TP) and 1 event was missed (FN). There were no alerts without recorded outbursts. According to the hospital, an average of 30 male patients at any one time in the ward was the basis for calculating ‘no-outburst/no-alert’ (i.e. ward was fully occupied but during the sessions patients were brought in, or taken out of, the ward to court, psychiatric evaluations, family meetings, etc). The frequency of outbursts was not similar among the patients. The True Negative (TN) cases, i.e. the number of predicted events in which there was ‘no-outburst/no-alert’ was calculated as follows: based on the hospital reports (Fig. 5) we assumed that each patient may develop one violent outburst per each session (=30 events, number of sessions is detailed in Table 2), we then subtracted out of 30 events the true positive (TP) cases, false-positive (FP) cases and false-negative (FN) cases of that session. For example the number of True Negative (TN) cases in a session with a dog alert which resulted in a true outburst in the ward (TP), and a dog alert but no outburst (FP), and an outburst in the ward without a dog alert (FN), was calculated as 27 TN cases. The number of TN cases from all sessions was summed. This calculation yielded a final value of 2206 TN cases during dog pre-puberty (Table S2, Fig. 2A). In a similar way to above, we calculated 286 TN cases during dog adulthood (Fig. 2B). Both ‘focused’ and ‘unfocused’ alerts and outbursts inside the ward were considered for this analysis (see definitions for ‘focused’ vs. ‘unfocused’ alerts at the Results section).

### Canine cortisol level

Samples of dog saliva were collected before a session once arriving to the hospital ground (18 samples, labelled ‘baseline’) or 20–30 min after alert within the ward (6 samples, labelled ‘alert’) and placed in the freezer of the hospital. Out of all samples, there were 3 pairs of samples (before/after alert) per each dog, a total of 6 pairs. The time point was selected based on a previous report showing that after stress, salivary cortisol level peak after this time point^24^. Samples were collected between June to September 2010. At the laboratory, samples were prepared according to the manufacturer’s instructions and assayed for of level of cortisol in saliva using Cortisol Saliva ELISA kit (SA E-6000, Labor Diagnostika Nord). The assay was performed in duplicate and the absorbance was read at 450 nm (Infinite, Tecan).

### Data integration and statistical analyses

The principal investigators integrated the data. Within the session, data were collected for any alert by each of the dogs. In parallel, data were collected for any outburst of the patients, monitoring the patients continued up to 24 hours following each session. The results of patients’ violent outbursts by dogs were analysed with GraphPad Prism (versions 5–7) using contingency 2 × 2 table with the following categorical variables: the number of correct ‘alert/outburst’ cases (True Positive (TP) cases), the number of ‘no alert/no outburst’ cases (True Negative (TP) cases, calculated as detailed above), the number of ‘no alert/outburst’ cases (False negative (FN) cases) and the number of ‘alert/no outburst cases (false positive (FP) cases). Chi square (pre-puberty) or Fisher’s exact test (adulthood) analyses, two-sided, with 95% confidence interval (CI), were used to compare categorical variables. In contingency analysis, n is the number of total frequency. Data were analysed anonymously and the patients’ ID altered during sessions (i.e. subjects were not necessarily repeatedly measured). The results of effect size were analysed with GraphPad Prism (version 7). All hypotheses were tested with two tails, *p* value < 0.05 was considered statistically significant.

The ‘effectiveness’ of the dog as an alert under repeated observations per animal was analysed using the Cochran’s Q test, IBM® SPSS® version 25. The test examined whether alert rates (‘alert’/’no alert’) changed over time (hours). Each dog was regarded as a ‘case’ with repeated measures (over the hours). For each dog, data were analysed from 152 hours in pre-puberty (total 304 hours for both dogs) and 18 hours in adulthood (total 36 hours for both dogs), see Table 1 (n is 170 hours per dog). For this analysis, repeated alerts were regarded as ‘alert’ only once and both focused and unfocused alerts were analysed. IBM® SPSS® treated ‘alert’ as a ‘success’. *p* value < 0.05 was considered statistically significant.

Results of ELISA assay for salivary cortisol level were analysed using the ‘One phase decay’ analysis, GraphPad Prism version 6. The sensitivity of the kit was 0.024 ng/ml (0.07 nM). One sample (‘baseline’ group) was below this level and therefore was excluded. Data were analysed with GraphPad Prism version 8. All samples were analysed using unpaired two-tailed Student’s *t*-test. Pairs of samples were analysed by two-way analyses of variance (ANOVA), each column represented a different outcome (baseline or alert) so matched values are spread across a raw, each raw represented a different day, so matched values are stacked into a subcolumn (by a dog). Data are expressed as means ± SEM. *p* < 0.05 considered statistically significant.

Mean physical restraints of patients per hour was calculated from raw data and analysed by column statistics (GraphPad Prism). Mean outbursts of patients (in ward) per hour was calculated from raw data and analysed by column statistics (GraphPad Prism). Results are expressed the means ± upper/lower CI, *p* < 0.05 considered statistically significant.

At each age, ratio of ‘focused’ or ‘unfocused’ dog alerts (numerator) out of total alerts (denominator) and the 95% CI of ratios were calculated using the Wald method (http://www.graphpad.com/quickcalcs/confInterval2/). Data are expressed as ratios ±95% CI. *p* < 0.05 considered statistically significant.

## Supplementary information

Bakeman et al-SUPPLEMENTARY INFO
