# Supplementary material for: Detection of Impending Aggressive Outbursts in Patients with Psychiatric Disorders: Violence Clues from Dogs
Source: Sci Rep. 2019 Nov 21;9:17228. doi: 10.1038/s41598-019-52940-w (PMC6872740; doi:10.1038/s41598-019-52940-w)
Supplement: Supplementary file 1 — Bakeman et al-SUPPLEMENTARY INFO [file 41598_2019_52940_MOESM1_ESM.docx]

Detection of Impending Aggressive Outbursts in Patients with Psychiatric Disorders: Violence Clues from Dogs

Uriel Bakeman^1,6^, Hodaya Eilam^2,6^, Clara Moray Schild^3^, Dan Grinstein^4^, Yuval Eshed^4^, Morris Laster^1^, Ester Fride^7^, Sharon Anavi-Goffer^5,6*^

^1^Mind Print Ltd., 7/3 Yair Stern St., Herzeliya, 46421, Israel;

^2^Unaffiliated, San Jose, California, USA;

^3^Forensic Psychiatry Inpatient Unit, Abarbanel Mental Health Center, Bat Yam, 59100, Israel;

^4^Yoad Eshed Ltd., Bet-Yehoshua, 40591, Israel;

^5^Institute of Medical Sciences, University of Aberdeen, Aberdeen, AB25 2ZD, UK;

^6^These authors contributed equally: Uriel Bakeman, Hodaya Eilam and Sharon Anavi-Goffer;

^7^Ester Fride is deceased.

^*^Author for correspondence:

Sharon Anavi-Goffer

Institute of Medical Sciences, University of Aberdeen, Aberdeen, UK

E-mail sharon.anavi-goffer@abdn.ac.uk

### Supplementary Information

### Supplementary Methods

### Training programme of violence-alerting dogs

Golden Retrievers are considered non aggressive, not territorial, patient, sensitive to odorants and easy to train. Two litter mate female pups underwent training to signal their owner of impending violent threats. Once they exhibited the ability to detect violent outbursts, the dogs were introduced to the hospital inpatient psychiatric ward for further training. The training continued throughout their life.

The research was conducted in two parts. Dogs were introduced to the hospital ward from age 2 months up to 1.5 years. The first section spanned 7 months during which time the dogs were pre-puberty (2 months - 1.5 years). The dogs were then kept at a special caring facility and returned at age 2 years to the hospital for a second round, when they were defined as adult (pre-trained and experienced). The reason for this was that the study was postponed due to the exchange of Principal Investigators (see Acknowledgments). As a result, we repeated for a shorter time the study to re-test the results from pre-puberty. We also tested if the dogs had maintained their alerting skills after such a long time with no practice. Due to the important implications of the results we decided not to combine the data and present the results of the two periods separately.

Because little is known about how neutering or the oestrus cycle affects canine alerting skills the dogs were not neutered and during the oestrus cycle the dogs did not work, with one exception, the three sessions in the closed room (see below) were performed when both dogs had their oestrus cycle (3, 6 and 13 days into their cycle, respectively). We found that during the oestrus cycle the dogs still alerted, however, a dedicated comparative study would be needed to address the level of differences under these conditions.

**Step 1:** Two Golden Retriever litter mate female pups were selected according to the ‘Tests of Campbell’ method [^1^](#_ENREF_1)^,^[^2^](#_ENREF_2). These tests are commonly used to assess the personality of puppies, scoring their level of dominance, submission, independence and socialization to humans [^3^](#_ENREF_3)^,^[^4^](#_ENREF_4). The following criteria were used for selection: social attraction, wailing, retrieving, restraint, response to pain, response to a foreign human, call for help, sensitivity to touch, sight and sound. In addition, the body structure of the dog was reviewed. In order to improve the selection of puppies for this study, the selection process was performed by an experienced dog trainer and the tests were performed for a total of four cycles. Finally, the temperament of the puppies that were selected for this study was moderate plus a tendency to rely on the leader, without presenting independent behaviour. Although the ‘Tests of Campbell’ are most commonly used to evaluate the physical appearance of a dog and to predict its personality at adulthood [^3^](#_ENREF_3), it has been suggested that this evaluation process can be improved by giving a numerical value for each type of answer [^4^](#_ENREF_4) or by adopting a modified set of stimuli and scoring protocol such as the Puppy Profiling Assessment [^5^](#_ENREF_5). A methodological characterisation of each behavioural test has been recently published and may help to improve this method in the future [^3^](#_ENREF_3). At the age of 6 weeks, the pups were separated from their mother and placed at a special caring facility for dogs. It has been documented that the earliest age to separate a weaned puppy from his mother and its puppy pack is six weeks, although some dog trainers believe that eight weeks is a better separating time. Importantly, it has been shown that pups that start to socialize with their owner at age of 5-9 weeks approach their owner more readily and are easier to train; some pups receive human socialization as early as 2 weeks [^6^](#_ENREF_6). Considering the puppy selection process, which tested their response to humans, we selected to start the training at age six weeks because at this age: (1) the puppies are still naive and sleep most of their time; (2) socialization processes start and it is easier for the trainer to direct their social interaction with humans; (3) the reinforcement of education training is easier.

**Step 2:** At the age of 6+ weeks, the pups underwent behavioural analysis tests and initial training to determine their ability for showing general alerting responses. Additional measurements at this stage included: disposition, control, distraction, responsiveness to human command, initiative, chase instinct, attention/sensitivity to handler, prolonged concentration, confidence, tolerance to children, energy/hyperactivity, attitude adaptability, dominance/self-interest, crate/kennel acceptance and complete veterinary evaluation of general health status. Specifically, the trainer taught the selected dogs various commands, using play, rewards and by praising them. Training focused on the common commands ‘come here’, ‘sit’, ‘down’ and ‘bark’. Most dogs bark as a means of communication, either to warn off potential intruders, sound an alarm, yelp in pain, express happiness, bay when chasing a prey and to express other feelings at different situations.

**Step 3:** From the age of 2+ months the animals underwent a 30 day obedience training program, during which preliminary obedience skills (common commands: sit, down, stay, halt) were instilled through an operant conditioning method of positive reinforcement reward (pet/ food/ happy tones). The idea of teaching a dog to bark on command is based on a common dog training procedure. Specifically, at first stage, in order to teach the dog to bark, the trainer frustrated it (e.g. give the dog to smell some good food but not allow it to eat). This was an individual training, because the dogs were not stimulated at the same rate. Some dogs need more vigorous stimuli while other dogs respond to light stimuli. At a second stage, after the trainer had trained the dog to bark on selected targets (e.g. a ball, food, a cat), the trainer started saying the command ’speak‘ before he wanted the dog to bark, and then praised and rewarded it when the dog responded appropriately. Some dogs get to this level after a single session and other need five or more sessions. Our trainer believes that it depends more on the dog’s temperament and not necessarily on its breed; i.e. some dogs will bark on command at around age of 6 weeks and others at age 2.5 months though they are all from the same breed.

Importantly, this training program is common and is not different from other routine training programs. Indeed training of alerting dogs for owners with diabetes or epilepsy is conducted by the trainer (UB) of this study in a similar manner, suggesting that for any selected condition, obedience of the dog to the owner is the key element. This element is based on the concept that the dog considers the owner as the ‘pack leader’ and will protect the leader of its pack against any harm. In this study, the trainer was also the owner/handler/’pack leader’ at the hospital. For other clinical indications, not in the scope of this study, the trainer gives a 3 weeks ‘control transfer’ training in order to forward the ‘pack leadership’ to a new owner. Leadership is generated by a very dominant body language during the training sessions and the dog gets food when it abides various hunting rules (e.g. when hunting as a pack each dog has a fixed position; obey the ‘leader’). In the capacity of a ‘leader’, the trainer is able to give food, take the food away, etc. This idea serves as the basis to handle the ‘leadership’ to a new owner.

**Step 4:** In addition, for this study, the dogs underwent response training such as training for specific barks on command and activation of responses to emergency situations. The training period included house breaking and the establishment of a mastering relationship with the dog (as described above). During training, the dogs learned to alert their master of any impending threats caused by a potentially violent person. For this the dog was presented, in the presence of the owner, to an ‘agitator’ who was acting as an intruder. The agitator presented a set of movements to the dog that are frequently preceded by agitation of patients with schizophrenia. These movements included: waving hands in aggressive ways, staring at the dog, sharp movements of the body and limbs, incorrect walking, in accordance with the list on Table S1. Thus the outcome was that the dog learned not to trust all people and be ready to protect its leader. Specifically, at this stage, the trainer taught the dogs to connect the command ‘speak’ with impending threats. Thus, the goal of the dog was to alert any danger associated with an impending attack on their master. At this stage the dogs still received a reward for correct response (e.g. petting).

**At the hospital:** Once the dogs reached the age of two months they were also introduced to the hospital inpatient psychiatric ward for further training. At this point, the dogs were trained to bark when a patient outburst occurred. Importantly, in practice, when this training step was successfully completed, the dogs started to alert on impending events (see Results and Table S4- most cases were alerted 30 minutes prior to event). This also means that the trainer did not use the command ‘speak’ thereafter. However, unexpectedly they spontaneously developed other types of communicating behaviour to interact with their trainer (see list in Table S3). At this point the experimental period began, but the training continued throughout their life outside the hospital. Importantly, there was no immediate reward at the hospital, specifically the trainer (‘pack leader’) did not give rewards during the experiment. This point is documented in the video linked to this manuscript. Importantly, there were cases in which the outbursts appeared some time, even hours, after the alerts, this further supports that the trainer had no reason to give a reward to the dogs. This also means that the dog alerts preceded the response of the staff at the wards.

### Behaviour questionnaire and evaluation of dog’s performance

The experiment began when both Golden Retriever dogs were introduced to the hospital ward from age 2 months up to 1.5 years and again at age 2 years. The number of observation days per month was similar between the two periods of the study (Table 2).

Their trainer was present at the ward for each session. Most sessions lasted 4 hours and only one session was conducted per day, after each session the dogs and owner left the hospital ground. Two dogs were used, each dog worked for 45 min alternating periods separated by periods of 1 hour and 15 min to rest and access to water. This break gave the dogs an opportunity to relax. The dogs were independent of each other as one dog was inside the ward and one dog stayed at the hospital garden area. During each session (except the sessions in the closed room), the trainer and the dog (on a leash) were located at a fixed position in the hall of the ward (see Figure 1 for exact location). The trainer sat on a chair during each work period (45 min) so that the trainer and the dog both had the same visual access to the patients, they were not separated from the patients and potentially could detect auditory and olfactory signals from the patients. Also the patients had visual access to the dogs but, though petting was not allowed, we cannot exclude the possibility that the dogs somehow influenced the behaviour of the patients. In many cases the dogs could not visualise subtle facial movements (cues) of the trainer. Furthermore, most outbursts occurred some minutes and even hours after the alerts (see Table S4), suggesting that in these cases the trainer would not be in a position to transmit specific cues to the dogs. There were also cases where the trainer was leaning forward to better visualise the far end of the hall. Also the dogs were held with a leash at all times. These movements and the tension of the leash could serve as cues, but the video film clearly shows that the dog did not alert or look to the other side of the hall in relation to the trainer leaning forward. Nonetheless, other subtle unknown cues may exist.

In addition, our report identified whether the alert was directed at a specific patient or not; these alerts were termed ‘focused’ and ‘unfocused’ alerts, respectively. As a control, at each session, an independent experimental observer (female) of our research team was positioned at the nurse station as shown in Figure 1 (a closed unit within the room). The observers had a visual view to the dogs and the owner but they were not familiar to the dogs. The observers filled out an independent report at each session which documented. Thus the principal investigator crossed-checked information on outbursts from three sources: medical staff, independent observers and the dog trainer.

The trainer was responsible for recording the dog’s behaviour on a template questionnaire during each working session, based on a range of vocalisation and specific behavioural patterns (Table S3 [^7^](#_ENREF_7)). A very similar change of behaviour/questionnaire was documented by another study which followed alert dogs and their owners, all are patients with diabetes Type I [^8^](#_ENREF_8). Importantly, this study reports that each dog has used an array of types of behaviour to alert, similarly to the observations we have found in our study. Thus, the types of dog behaviour observed in our study replicates findings of previous studies and supports the hypothesis that each alert dog can adopt a variety of types of behaviour, regardless the training [^8^](#_ENREF_8)^,^[^9^](#_ENREF_9). In order to validate the accuracy of the trainer report we videoed the dogs’ behaviour at adulthood (when the dogs were positioned in the hall). Their behaviour, alert dates and times were then compared with the trainer reports.

An additional control was conducted where the trainer and the dogs were re-positioned to a closed room (with a closed window), adjacent to the hall; the door was closed so there was no visual access to the patients. This control was conducted for three sessions when the dogs were adult. The results were not included in our analysis (observations from hall of the ward) but are reported in the Results section.

### Video film of the alert dogs

Link to download the video film

[https://figshare.com/s/b93110bd8b61fe61a9b7](https://urldefense.proofpoint.com/v2/url?u=https-3A__figshare.com_s_b93110bd8b61fe61a9b7&d=DwMF-g&c=vh6FgFnduejNhPPD0fl_yRaSfZy8CWbWnIf4XJhSqx8&r=AGZMqGQLHtqhSvnhw-pkFVF74VU1eYzNL3T3zwVRDOk&m=pjMBLTMG8tbSiB7gZy0Y9g428AKCXApey36o-mFZRLA&s=OZuZ24CWJFAUX2VdoFOSHUTB0LrJaGsr0Nu-e2lRI04&e=)

### Supplementary Tables

### Table S1.

### Categories of violent activity

| **Category** | **Example behaviours** |
| --- | --- |
| Verbal violence | Yelling, cursing, direct or indirect threat |
| Physical violence towards objects | Door slamming, breaking or throwing of objects |
| Self-inflicted violence | Scratching, slamming into walls, self inflicted cuts. |
| Physical violence toward others | Threatening gestures, biting, kicking, hitting |

### Table S2.

### Contingency at dog pre-puberty

|  | **Outburst** | **No outburst** | **Total** |
| --- | --- | --- | --- |
| **Alert** | 56 (TP) | 9 (FP) | 65 |
| **No alert** | 10 (FN) | 2206 (TN) | 2216 |
| **Total** | 66 | 2215 | 2281 |

‘Outburst’, refers only to outbursts inside the ward (visual space of the dog). ‘Alert’ refers to ‘focused alerts’. True Positive (TP): Violent outbursts which were alerted by the dogs. False Negative (FN): outbursts which were missed, i.e. the dogs did not alert true outbursts. False Positive (FP): Alerts without recorded outbursts, i.e. there were no violent outbursts up to 24 hours after changes in dogs’ behaviour were recorded. True Negative (TN) cases are the number of predicted events in which there was ‘no-outburst/no-alert’. *** p<0.0001 (Chi-square, 1651, df =1).

### Table S3.

### Characterisation of the dog behaviour at different ages

| **Dog behaviour** | **Pre-puberty** | **Adult** |
| --- | --- | --- |
| Staring a patient- ‘focused’ alert | **+** | **+** |
| Staring the master | + | + |
| Unsettled movements | + | - |
| Posture- tensed | + | - |
| Posture- immobile/ motionless | + | - |
| Licking the master hands | + | + |
| Chewing on staff nervously | + | - |
| Barking/Howling/ Growls | + | - |
| Sharp movements | - | + |
| Sharp movements- side to side | - | + |
| Rotation | - | + |
| Pull the leash | - | + |

Classifying dog behaviour was based on previous studies [^7^](#_ENREF_7)^,^[^10-12^](#_ENREF_10). We have noticed that over time the dogs changed their characteristic behaviour. The master has learnt to differentiate actual alerts from other similar-yet-typical behavioural patterns exhibited by dogs such as attention-seeking. At pre-puberty, the dogs behave similarly and tended to adopt immobile/motionless postures (85%) or used vocals (bark/howl/growl, 92%) or stared at a patient (78%) in order to alert and to attract the attention of their master. In adulthood, both dogs had common behavioural patterns including staring at a patient or at the master, and licking the master’s hand.

### Table S4.

### Relationship between the time that the dog altered and the actual outburst

| **Time range** | **Specific time difference between alert and outburst** | | | | **No. alerts** |
| --- | --- | --- | --- | --- | --- |
| 0-10 min | 00:07 | 00:05 | 00:05 | 00:04 | 4 |
| 10-30 min | 00:12 | 00:20 |  |  | 2 |
| 30-60 min |  |  |  |  | 0 |
| 60-120 min | 01:10 |  |  |  | 1 |
| 2-4h | 02:22 |  |  |  | 1 |
| 4-12h |  |  |  |  | 0 |
| 12-24h | 12:53 |  |  |  | 1 |

Nine such events were recorded during dog adulthood. The difference in time from the first alert to the outburst was calculated. Repeated alerts were excluded from the analysis.

### Table S5.

### The ‘violent’ patient is identified by different dogs

| **Session** | **Dog** | **Time** | **Type of alert** | **Time of the alert** |
| --- | --- | --- | --- | --- |
| 1 | Buby | 15:00- 15:45 | Non | - |
| 2 | Bambi | 15:45-16:30 | Non | - |
| 3 | Buby | 16:30-17:15 | ‘Focused’ alert at patient #1 | 17:08 |
| 4 | Bambi | 17:15-18:00 | ‘Focused’ alert at patient #1 | 17:21 |

### Table S6.

### The same dog alerts several times the same patient with a violent outburst

| **Session** | **Dog** | **Time** | **Type of alert** | **Time of the alert** |
| --- | --- | --- | --- | --- |
| 1 | Buby | 15:23- 16:13 | Rotate, unsettled | 15:23 |
|  |  |  | Unfocused alerts | 15:24-15:36 |
|  |  |  | Dog settles | 15:38 |
|  |  |  | Unsettled, focused alert on patient #2. | 15:50-15:58 |
|  |  |  | Patient #2 is moving away. Dog stayed tensed. | 16:01 |
|  |  |  | Rotate, unsettled, alert is focused on patient #2 | 16:03-16:05 |
|  |  |  | Patient #2 is moving away. Dog stayed tensed. | 16:06 |
| 2 | Bambi | 16:15-16:55 | Focused alert on patient #2 | 16:21-16:22 |
|  |  |  | Patient #2 is moving away. Dog settles. | 16:23 |

### Supplementary References

1 Campbell, W. E. 137–144 (American Veterinary Publications, California, 1975).

2 Diederich, C. & Giffroy, J.-M. Behavioural testing in dogs: A review of methodology in search for standardisation. *Applied Animal Behaviour Science* **97**, 51-72, doi:10.1016/j.applanim.2005.11.018 (2006).

3 McGarrity, M. E., Sinn, D. L. & Gosling, S. D. Which personality dimensions do puppy tests measure? A systematic procedure for categorizing behavioral assays. *Behavioural Processes*, doi:10.1016/j.beproc.2014.09.029 (2014).

4 Perez-Guisado, J., Munoz-Serrano, A. & Lopez-Rodriguez, R. Evaluation of the Campbell test and the influence of age, sex, breed, and coat color on puppy behavioral responses. *Can J Vet Res* **72**, 269-277 (2008).

5 Asher, L. *et al.* A standardized behavior test for potential guide dog puppies: Methods and association with subsequent success in guide dog training. *Journal of Veterinary Behavior: Clinical Applications and Research* **8**, 431-438, doi:10.1016/j.jveb.2013.08.004 (2013).

6 Serpell, J. & Jagoe, J. A. Early experience and the development of behaviour. pp. 79–102. In: The Domestic Dog: Its Evolution, Behaviour and Interactions with People (Serpell, J. ed.), Cambridge University Press, Cambridge. (1995).

7 Wells, D. L. Dogs as a diagnostic tool for ill health in humans. *Altern Ther Health Med* **18**, 12-17 (2012).

8 Rooney, N. J., Morant, S. & Guest, C. Investigation into the value of trained glycaemia alert dogs to clients with type I diabetes. *PLoS One* **8**, e69921 (2013).

9 Dalziel, D. J., Uthman, B. M., McGorray, S. P. & Reep, R. L. Seizure-alert dogs: a review and preliminary study. *Seizure* **12**, 115-120 (2003).

10 Di Vito, L. *et al.* A seizure response dog: video recording of reacting behaviour during repetitive prolonged seizures. *Epileptic Disord* **12**, 142-145 (2010).

11 Kirton, A., Winter, A., Wirrell, E. & Snead, O. C. Seizure response dogs: evaluation of a formal training program. *Epilepsy Behav* **13**, 499-504 (2008).

12 Tauveron, I., Delcourt, I., Desbiez, F., Somda, F. & Thieblot, P. Canine detection of hypoglycaemic episodes whilst driving. *Diabet Med* **23**, 335 (2006).
